# Palaeoclimate has a major effect on the diversity of endemic species in the hotspot of mountain biodiversity in Tajikistan

**DOI:** 10.1038/s41598-021-98027-3

**Published:** 2021-09-21

**Authors:** Małgorzata Raduła, Sebastian Świerszcz, Marcin Nobis, Sylwia Nowak, Agnieszka Nobis, Arkadiusz Nowak

**Affiliations:** 1 Botanical Garden, Center for Biological Diversity Conservation, Polish Academy of Sciences, Prawdziwka 2, 02-976 Warszawa, Poland; 2 Department of Ecology, Biogeochemistry and Environmental Protection, University of Wrocław, Kanonia 6/8, 50-328 Wrocław, Poland; 3 Institute of Biology, University of Opole, Oleska 22, 45-052 Opole, Poland; 4 Faculty of Biology, Jagiellonian University, Gronostajowa 3, 30-387 Kraków, Poland; 5 Research Laboratory ‘Herbarium’, National Research Tomsk State University, Tomsk, 634050 Russia

**Keywords:** Biodiversity, Biogeography, Climate-change ecology, Conservation biology, Biodiversity, Biogeography, Climate-change ecology, Conservation biology

## Abstract

In a period of ongoing climate changes, identifying drivers of overall and endemic species diversity is a key element in constructing new ecological patterns and determining the main goals of conservation. Such studies are especially crucial if they concern biodiversity hotspot areas. In this study, we explore patterns and drivers of plant endemism (the proportion of endemic plant species to overall plant species richness; PE) in Tajikistan. We used three groups of climatic measures featuring the contemporary and glacial climates as well as climatic changes since the Last Glacial Maximum in the Pleistocene (LGM). To explore relationships between PE and climatic groups, and the most important climatic variables, we applied the Generalised Additive Model and regression trees method respectively. Glacial climate predicted PE variation the most (74.3%), followed by climate stability (55.4%) and current climate (62.4%). The most important variables represented change in precipitation of driest quarter, glacial mean annual temperature and current annual precipitation. LGM climate and its change to date have the greatest influence on contemporary PE patterns in Tajikistan, revealing the evolutionary dependencies between limited-range plants and past climate. Accordingly, annual temperature and precipitation regimes have been the most crucial drivers of PE since the LGM until today. The study revealed the dependence of the PE on a stabilized water-energy supply. The changing temperature and precipitations regimes during the ongoing climate warming may, therefore, increases the threat to geographically isolated cryophilous plants of Tajikistan, while their escape potential to suitable cold habitats is highly topographically limited.

## Introduction

Tajikistan is a mountainous Central Asian country, located almost entirely within the Pamir-Alai Mountains (Mts). With one of the largest altitudinal amplitudes in the world, diverse geology, considerable glacier cover, extreme precipitation and temperature fluctuations, the area of Tajikistan conducives a great number of plant species and plant communities. The landscape of the country is relatively young, resulting from the recent Cenozoic uplift that shaped the sharp crests of the Pamir-Alai and Tian-Shan Mts. These are a part of the long orogenic belt of Asia along the western section of the Himalaya, Karakorum and Hindukush ranges^1^. There are large and wide, often geographically isolated valleys with gentle slopes in the colline and montane belts that have not been glaciated and play the role of refuge sanctuary for Pleistocene or Tertiary species^2^.

These unique environmental conditions promote a high rate of endemism. Out of ca. 4,300 vascular plants naturally occurring in Tajikistan, almost 1,500 are endemics (sensu stricto and subendemics), which is equal to ca. 35% of the total flora of Tajikistan^3,4^. There are 12 endemic and 14 subendemic genera in the country. To the richest genera belongs *Astragalus*, comprising 173 endemic species, with its exceptional richness probably related to niche diversification in the middle to late Pleistocene^5^.

Because of its floristic richness, Tajikistan is recognized by Conservation International as a hotspot of biodiversity and one of the eleven most important focal points for conservation planning and future plant diversity studies^6^. Simultaneously, the country is regarded as the most susceptible region to climate change and biodiversity loss worldwide, but still only twelve species from this country are listed as globally endangered (e.g. Darvaz dogwood *Swida darvasica* and wild apple *Malus sieversii*)^4^. Recent analysis of the degree of the endangerment of Tajik flora shows considerable threats to its richness, where 1,627 taxa (38.11% of all native species) are threatened and 23 extinct (0.54%)^4^.

In Tajikistan, the Pleistocene glaciers in the Pamir and the Alai–Turkestan ranges have been valley glaciers, except for the glaciers on the Pamirian Plateau, which have formed local piedmont glaciations. In the deeper valleys the vegetation survived, and after developing a woodland vegetation these harbour a number of old lineages, e.g. *Ostrowskia magnifica*. The Pleistocene glacier advances all over western High Asia were contemporaneous with climatic cold phases rather than monsoonal maxima. Aridity increased over the region after changes in the precipitation patterns and an increase in the influence of westerly circulation and the Siberian anticyclone. Only in some ranges of south-eastern Pamir (e.g. Shahdarian Mts., Wakhan Mts.) is it likely that indirect monsoonal influence may have been responsible for the existence of the Late Glacial moraine stages in this area^7^.

The Pleistocene glaciation significantly influenced vegetation and the current distribution patterns of species. It is commonly accepted that the contemporary diversity patterns of endemism are the results of past processes of speciation and extinction^8–11^. Therefore, they not only reflect the influence of the contemporary climate on species ranges, but are obviously strongly related to past climate regimes and their oscillations (e.g.^12^). Regions with long-lasting speciation due to climatic changes or stability, as well as biogeographical or landscape circumstances, evolve a rich proportion of endemic flora. In contrast, regions with a high rate of extinction are poor with a restricted species range^8^. Therefore Tajikistan, a relatively small country in the centre of Asia, has experienced various climatic changes while at the same time buffering these in some locations, giving the chance for both paleoendemics to be retained and neoendemics to arise. However, despite the extraordinary uniqueness of vascular flora in Tajikistan, the patterns of distribution and processes behind today’s endemic species richness are poorly understood.

Long-term climatic changes affect the regional species pool, the evolutionary response of plant species and species migrations during glacial-interglacial fluctuations, which could be particularly pronounced in topographically diversified Tajikistan^13,14^. The Stability-Time-Hypothesis or Climate-Stability-Hypothesis can be tested in the valleys^8,13^ and a contradicting hypothesis of speciation in young unfertile landscapes can be checked as well^15^. Regions of high long-term spatio-temporal climatic stability are expected to be rich in plant species (including endemics species) due to long-term speciation processes^13^. The specific landlocked location of Tajikistan has to be considered in the context of the most recent climatic changes. As the influence of large water bodies (e.g. oceans, seas or great lakes) plays no role in the region, climatic instabilities such as Milankovitch climate oscillations^13^ cannot be buffered by the environment. This increases the extremities of the mountainous, topographically diverse environment, make it extraordinarily harsh. Most of Tajikistan is also characterised by high environmental heterogeneity. Diversified topography and geology surely stimulate species speciation (including endemics) and its survival during unfavourable changes. This survival is possible, as in other mountainous countries, due to the persistence of a number of micro-refuges that have relatively stable conditions and can mitigate the effects of climatic extremes^16^.

The aim of this study is to investigate patterns of plant endemism in Tajikistan in relation to contemporary and glacial (Last Glacial Maximum, LGM) climate as well as climatic stability (reflected by the differences between current and glacial climatic variables). We hypothesize, that the contemporary endemism of Tajikistan is mostly dependent on the past climate regime and its instability till present time. The specific questions we addressed to are the following: (1) which climatic variable set (glacial, current or climatic stability) explains endemism patterns the best for the Pamir-Alai Mts. within Tajikistan?; (2) what are the most powerful climatic drivers of endemism in in this area?; and (3) which mountain ranges are the most important refuges for the endemic plants of Tajikistan?

## Results

**Figure 1:**
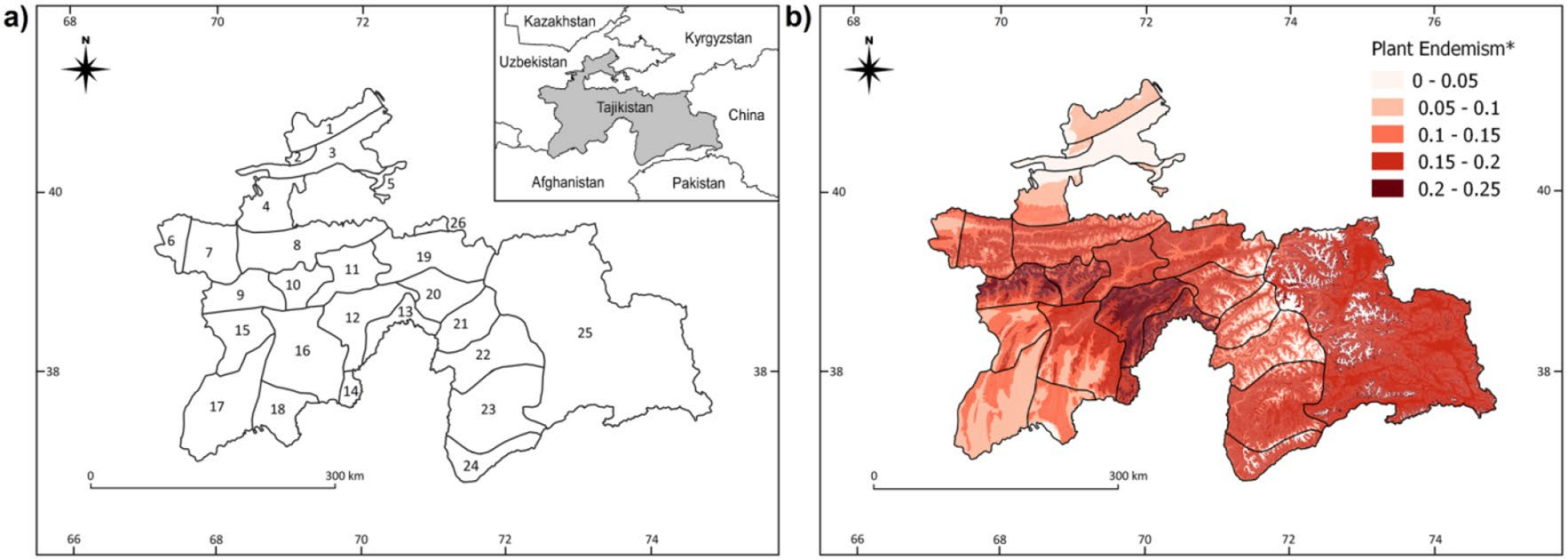
Map of phytogeographical subregions (**a**), and the distribution of the plant endemism (*proportion of endemic plant species to overall plant species richness) in Tajikistan (**b**). Subregions: 1—Kuraminian, 2—Mogoltausian, 3—Prisyrdarian, 4—Turkestanian A, 5—Turkestanian B, 6—Zeravshanian A, 7—Zeravshanian B, 8—Zeravshanian C, 9—Hissar-Darvasian A, 10—Hissar-Darvasian B, 11—Hissar-Darvasian C, 12—Hissar-Darvasian D, 13—Hissar-Darvasian E, 14—Hissar-Darvasian F, 15—South Tajikistan A, 16—South Tajikistan B, 17—South Tajikistan C, 18—South Tajikistan D, 19—East Tajikistan A, 20—East Tajikistan B, 21—East Tajikistan C, 22—West Pamirian A, 23—West Pamirian B, 24—West Pamirian C, 25—East Pamiraian, 26—Alaian. Maps were created using QGIS v. 2.18 (Quantum GIS, https://www.qgis.org).

After verification of the endemic status of Tajik plants, we compiled in our model data on the occurrence of 4269 species, with 1243 endemic to the country. The highest proportions of endemics were found on Hissar and Darvaz Mts. (Fig. 1b). The GAMs summary and parameters for climatic variables are shown in Table 1.

**Table 1:** Results of generalised additive models (GAM) of the most important climatic variables in relation to proportion of endemic plant species to overall plant species richness (PE).

	Current climate			LGM climate			Climate stability		
	edf	F	*p*-value	edf	F	*p*-value	edf	F	*p*-value
Annual mean temperature [C°]	6.83	57.41	< 0.001	7.05	88.18	< 0.001	7.13	21.43	< 0.001
Isothermality [%]	4.65	5.92	< 0.001	8.51	22.13	< 0.001	6.11	11.48	< 0.001
Annual precipitation [mm]	6.89	7.02	< 0.001	7.77	18.48	< 0.001	3.64	16.44	< 0.001
Precipitation seasonality [%]	6.79	14.90	< 0.001	7.24	22.02	< 0.001	1.95	1.37	n.s
Precipitation of the driest quarter [mm]	6.68	13.64	< 0.001	5.57	13.90	< 0.001	6.40	38.61	< 0.001
	Adj. R^2^ = 0.608			Adj. R^2^ = 0.73			Adj. R^2^ = 0.539		
	Dev. expl. = 62.4%			Dev. expl. = 74.3%			Dev. expl. = 55.4%		

Three groups of climatic measures were used in three separate models: current climate, LGM climate, and climate stability (climatic change since the LGM).

*LGM* Last Glacial Maximum in the Pleistocene, *edf* effective degrees of freedom, *Adj. R*^*2*^ Adjusted R-squared, *Dev. expl.* deviance explained.

**Figure 2:**
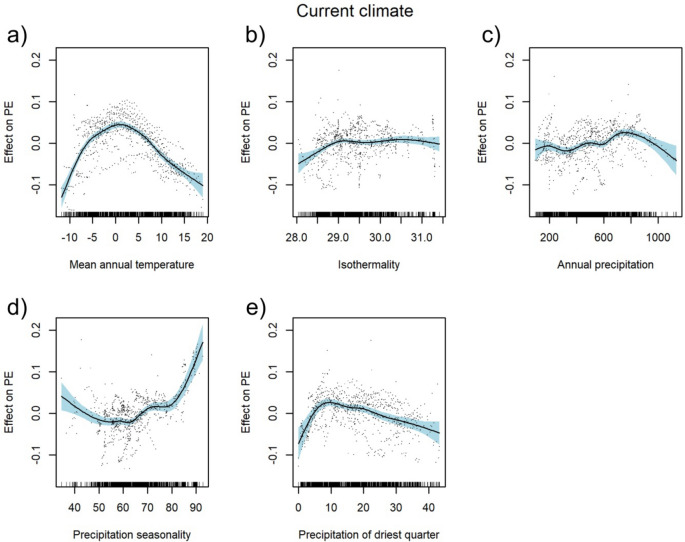
Partial response curves of current climate predictors with smooth effects in the generalised additive model (GAM). Effect of annual mean temperature [°C] (**a**), isothermality [%] (**b**), sum of annual precipitation [mm] (**c**), precipitation seasonality [%] (**d**), and precipitation of the driest quarter [mm] (**e**); PE: proportion of endemic plant species to overall plant species richness as target variable. For model summary of the GAM see Table 1.

The current climate model explained 62.4% of the total variation. A hump-shaped relationship was identified between PEs and mean annual temperature with maximum diversity between − 2.5 and 5 °C (Fig. 2a). Isothermality showed a slightly increasing trend up to 29% with a stabilisation above this value (Fig. 2b). PEs slightly increased with the increasing sum of annual precipitation and, depending on geographical location, with fluctuation between 200 to 800 mm, and a decrease towards higher values (Fig. 2c). Precipitation seasonality at the beginning decreased slightly, achieving a minimum at ca. 60 mm and then steeply increasing above the value of 80 mm (Fig. 2d). On the other hand, PEs sharply increased with the increasing sum of precipitation in the driest quarter up to the value of around 10 mm and then gradually decreased with a dropping tendency of precipitation (Fig. 2e). Regression tree analysis revealed that, of all the current climatic variables investigated, the mean annual temperature and sum of annual precipitation had the highest relative impact on the PEs pattern (Fig. 5a). This analysis provides a regression tree with five splits resulting in six groups classified by these variables. The highest PEs (0.19) were noticed in areas with a sum of annual precipitation above 643 mm and a mean annual temperature above − 1.5 °C.

**Figure 3:**
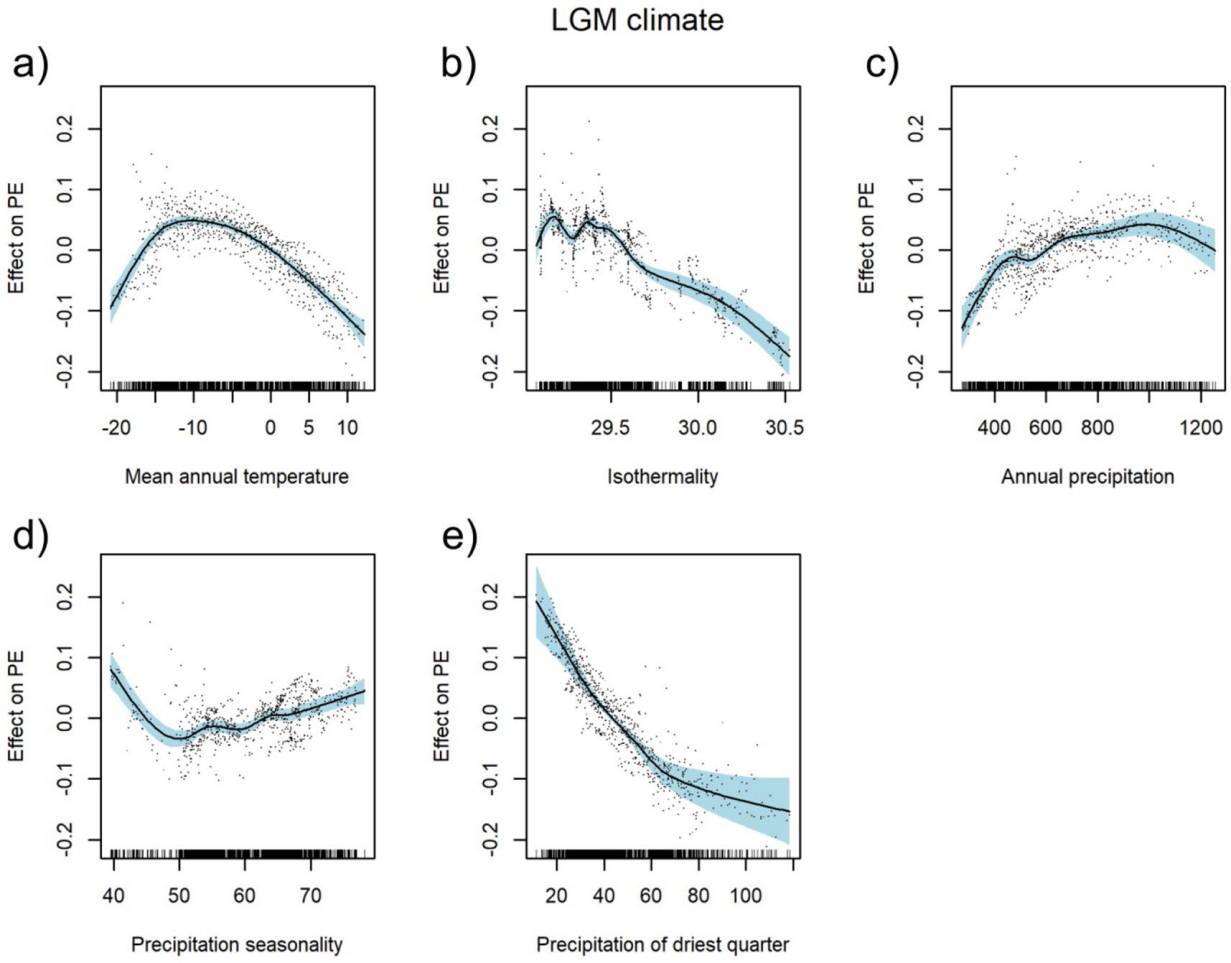
Partial response curves of Last Glaciation Maximum (LGM) climate predictors with smooth effects in the generalised additive model (GAM). Effect of annual mean temperature [°C] (**a**), isothermality [%] (**b**), sum of annual precipitation [mm] (**c**), precipitation seasonality [%] (**d**), and precipitation of the driest quarter [mm] (**e**); *PE* proportion of endemic plant species to overall plant species richness as target variable. For model summary of the GAM see Table 1.

The LGM climate model achieved an explained deviance of 74.3%. The relationship between PEs and quaternary mean annual temperature in the LGM was hump-shaped, with the peak between − 14 and − 5 °C (Fig. 3a). PEs decreased with increasing isothermality and sum of precipitation in the driest quarter (Fig. 3b,e) and increased with the increasing sum of annual precipitation (Fig. 3c). PEs steeply decreased with increasing precipitation seasonality up to ca. 50% and then slightly increased (Fig. 3d). Regression tree analysis revealed that, of all the LGM climatic variables investigated, the sum of precipitation in the driest quarter and the mean annual temperature had the highest relative importance for PE patterns (Fig. 5b). This analysis created a regression tree with three splits resulting in four groups with regard to these parameters. The highest PEs (0.16) was noticed in areas with a sum of precipitation in the driest season below 60 mm and a mean annual temperature below 4.4 °C in the LGM (Fig. 5b).

**Figure 4:**
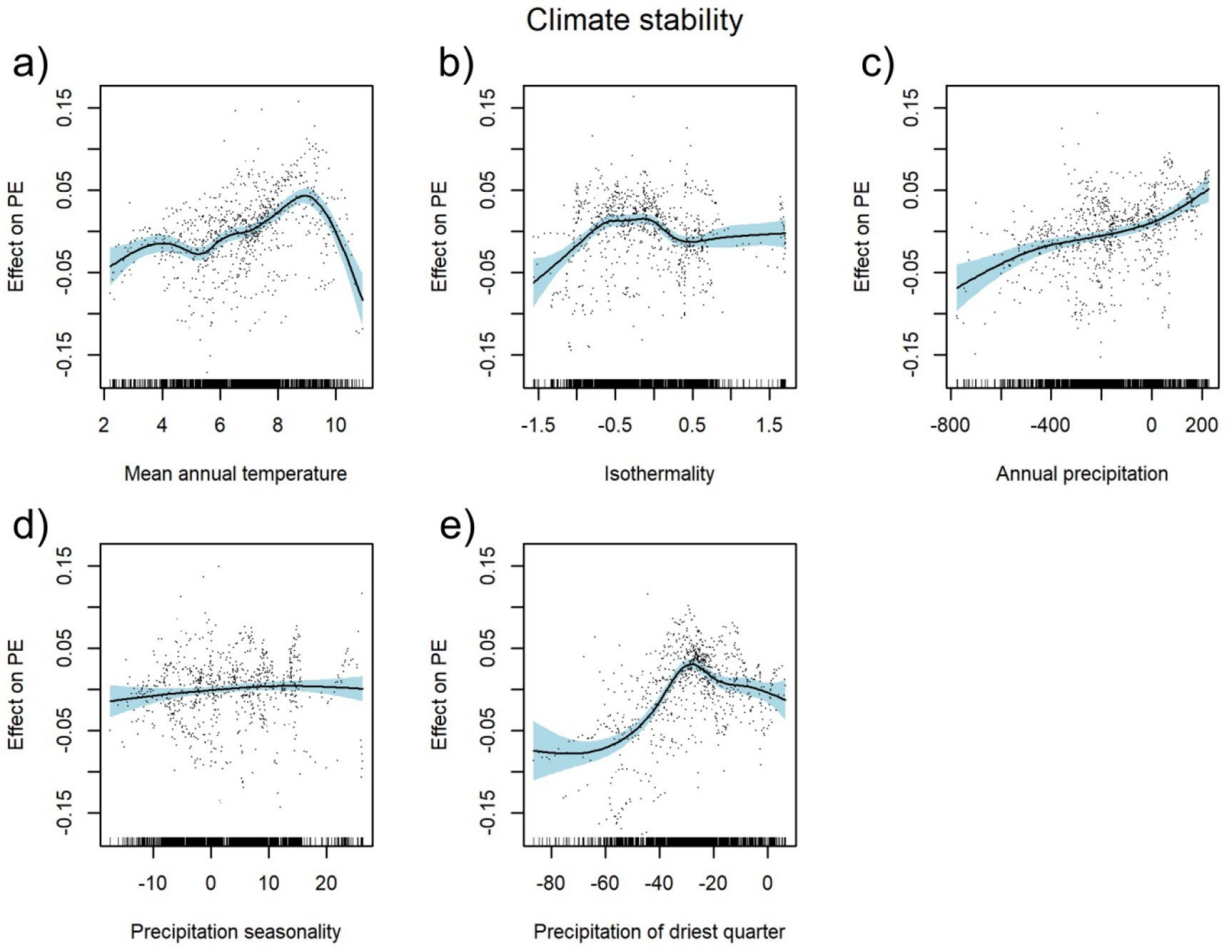
Partial response curves of climatic change predictors with smooth effects in the generalised additive model (GAM). Effect of annual mean temperature [°C] (**a**), isothermality [%] (**b**), sum of annual precipitation [mm] (**c**), precipitation seasonality [%] (**d**), and precipitation of the driest quarter [mm] (**e**); PE: proportion of endemic plant species to overall plant species richness as target variable. For model summary of the GAM see Table 1.

**Figure 5:**
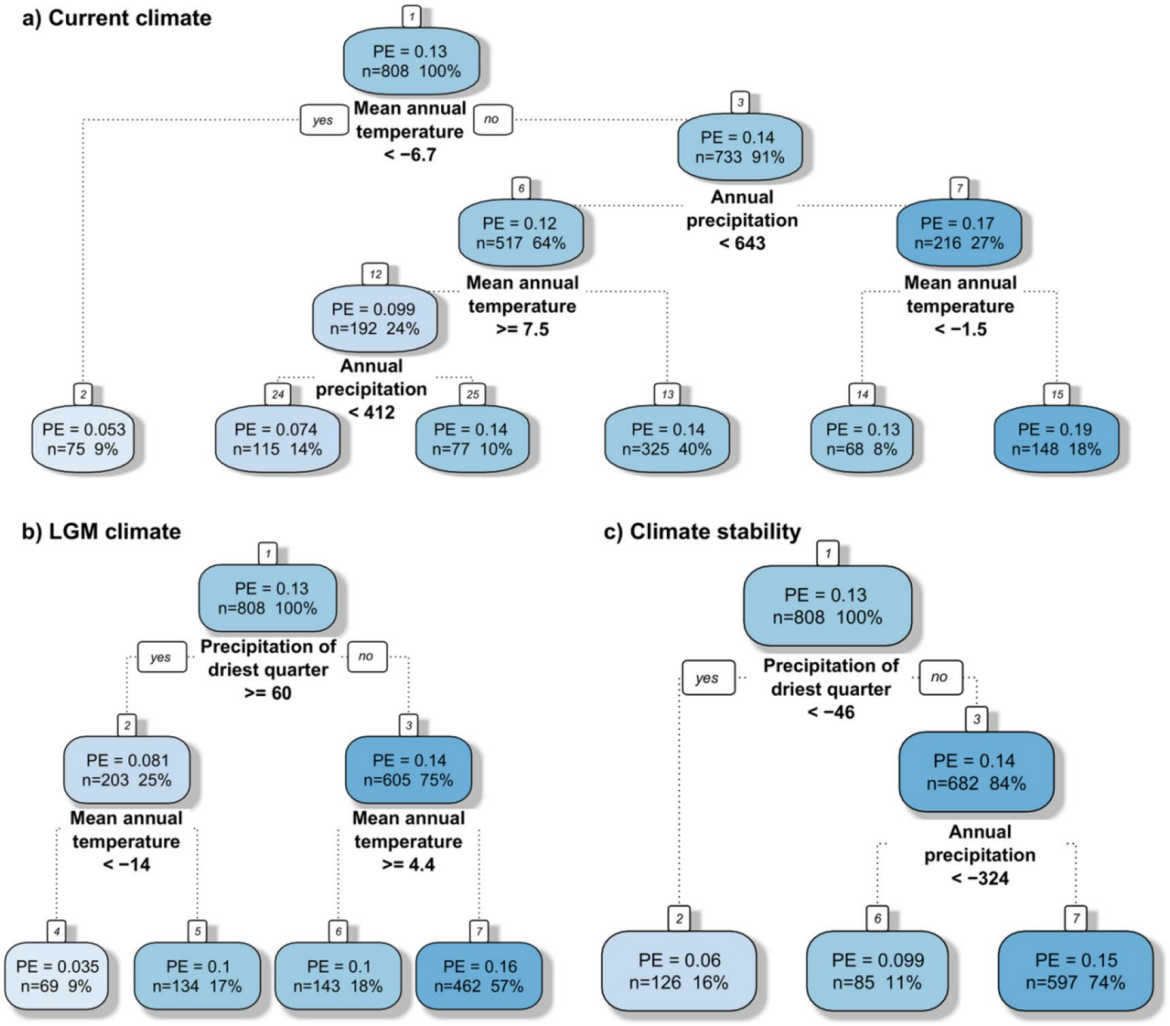
Regression trees of the most important climatic variables computed as predictors in relation to proportion of endemic plant species to overall plant species richness (PE). Three groups of climatic measures were used in three separate trees: current climate (**a**), glacial climate (**b**), and climatic change since the Last Glacial Maximum (**c**). Each splitting node (splitting variable name and splitting criterion) and each terminal node shows the mean PE, number and percentage of cases in the dataset.

The climate stability model computed for the PEs in Tajikistan explained 55.4% deviance. The PEs showed a ambiguous trend (first increasing, then decreasing) for mean annual temperature, isothermality and the sum of precipitation in the driest quarter, with the peak for mean annual temperature at around 9 °C (Fig. 4a), for isothermality between − 0.5 °C and 0 °C (Fig. 4b), and for the sum of precipitation in the driest quarter around − 30 mm (Fig. 4e). PEs increased with the increasing sum of annual precipitation between the current and LGM climate (Fig. 4c). Precipitation seasonality had a non-significant effect on PE (Table 1, Fig. 4d). Regression tree analysis revealed that, of all climate stability variables investigated, the difference in the sum of precipitation in the driest quarter and annual precipitation had the highest relative importance for the PE pattern (Fig. 5c). This analysis constructed a regression tree with two splits resulting in three groups with regard to these factors. The highest PEs (0.15) were noticed in areas with a decrease between the current and LGM sum of precipitation in the driest quarter higher than 46 mm and a total sum of precipitation above 324 mm.

**Figure 6:**
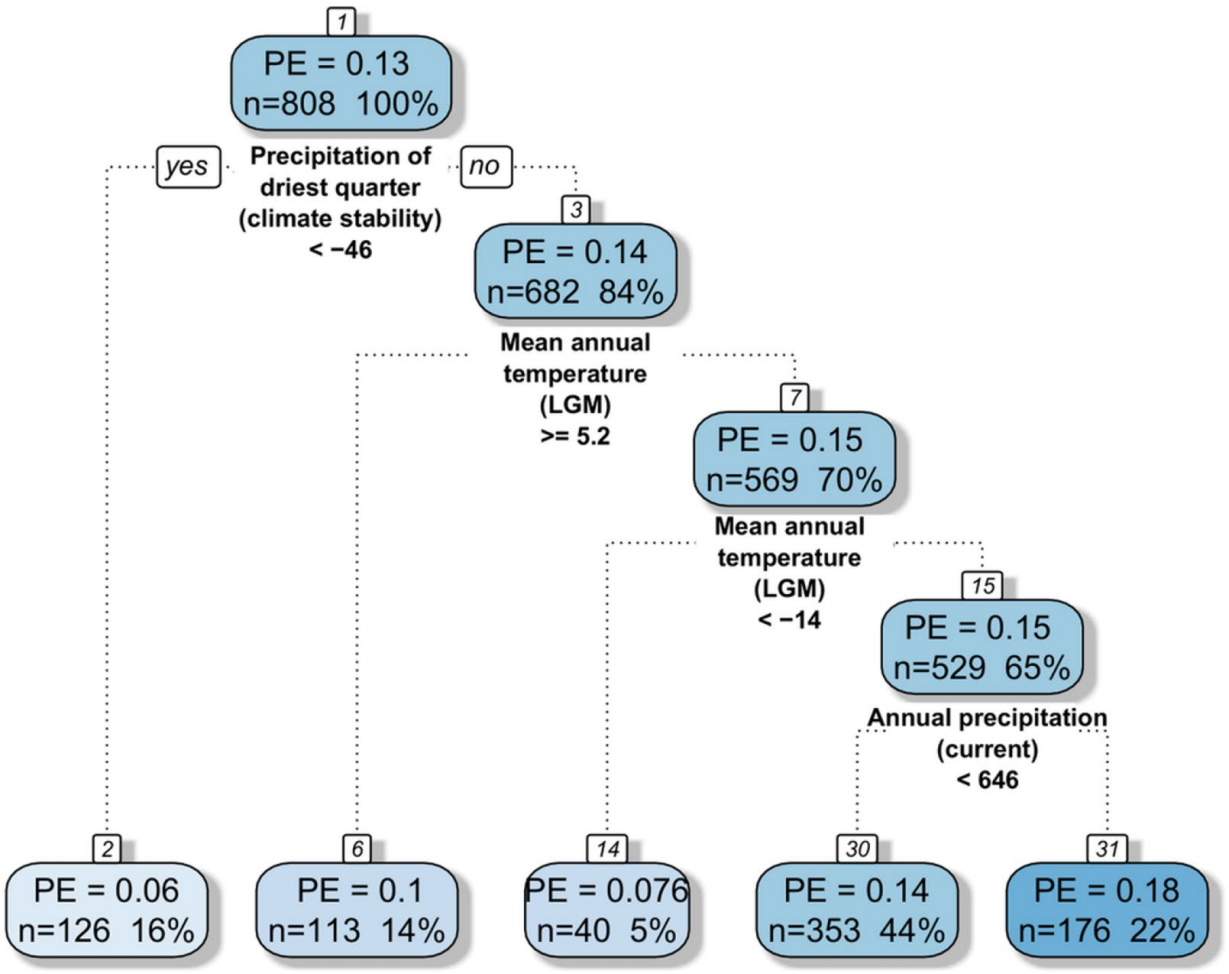
Regression tree of all climatic variables (current climate, glacial climate, and climatic change since the Last Glacial Maximum) computed as predictors in relation to proportion of endemic plant species to overall plant species richness (PE). Each splitting node (splitting variable name and splitting criterion) and each terminal node shows the mean PE, number and percentage of cases in the dataset.

Regression tree analysis, containing all bioclimatic variables (current climate, LGM climate and climate stability), revealed that the highest impacts on PE patterns were due to anomalies in the sum of precipitation in the driest quarter, mean annual temperature in the glacial and current sum of precipitation (Fig. 6). This analysis provides a regression tree with four splits resulting in five groups. The most important variable was the change of precipitation in the driest quarter, which indicated the highest PEs in areas with a decrease of more than 46 mm. The second most important variable was the mean annual temperature in the LGM, responsible for the second and third splits, indicating higher PEs in areas with an average annual temperature between − 14 and 5.2 °C in the LGM. The last split is linked to the current sum of precipitation, however it divides our data into two fairly similar groups (PE 0.14 and 0.18) with higher endemism in the wetter areas.

## Discussion

Tajikistan is characterised by diverse geomorphology and one of the largest altitudinal amplitudes in the world, having at the same time an extreme number of endemic species including both paleoendemics and neoendemics. Additionally, the unique history of its glaciation in the Pleistocene, the massive orogenesis in the Cenozoic, and the extraordinary influences of moderate, subtropical and Mediterranean climates have contributed to the uniqueness of the vascular flora of this territory. Nonetheless, in this relatively small region of Central Asia patterns of endemism and factors influencing them remain poorly understood.

### The Pleistocene climate is the most prominent driver of today’s endemic plant diversity in Tajikistan

Paleoclimate data are considered relevant for the explanation of the biogeography and ecology of endemism, however the detailed relations are still far from being fully understood^12^. In mountainous regions and in higher latitudes, glaciation in the Pleistocene in particular profoundly influenced species richness and endemism^17^. Past climate changes and glaciation cycles of the Pleistocene in Central Asia have surely led to significant changes in today’s distribution patterns of endemic species. Many of them have survived in the Pamir-Alai Mts in deep, wide valleys (e.g. *Ostrowskia magnifica*, *Cicerbita rosea*, *Corydalis nudicaulis*, *Exochorda korolkowii*, *Potentilla kulabensis*, *Pyrus lindleyi*, *Ranunculus chodzhamastonicus* and others) or on steep rock faces in the montane belt (e.g. *Fumariola turkestanica*, *Sergia regelii*, *Anemone seravschanica*, *Asperula laevis*, *Campanula hissarica*, *Dionysia involucrata*, *Kudrjaschevia nadinae*, *Scutellaria baldshuanica*, *S. megalodonta* and many others). All of them have withstood the changing climatic conditions and adapted to harsh, but relatively stable conditions in valley bottoms and on rock faces and have thrived in these habitats until now. The same processes have been observed in the Alps and other mountainous areas^18,19^ and reflect the long lasting processes of speciation and extinctions overridden by glacial cycles^8^. The Last Glacial Maximum (LGM) advanced the glaciers in the valleys of Tajikistan down to ca. 2500–2700 m a.s.l., so ca. 650–1000 m lower than today^7^. This explains why the peak of current endemism is at the subalpine belt between 1800 and 2800 m and then drops steeply with increasing elevation^3,4^.

Cenozoic orogenesis had a major impact on the evolution of the alpine flora in Tajikistan and other mountainous regions of southern Asia^20^. The wide uplift range of the Alborz and Zagros Mts. together with high fragmentation and isolation of their habitats are considered as important factors of high-elevation endemism^21^. On the other hand, similar climatic conditions during the Pleistocene glacial periods in the Himalayas, Central Asia, Iranian Caucasian, and some European mountain ranges enabled long-distance migrations of cryophilous species. Inter-glacial mitigation of climatic conditions created ecological barriers for cryophiles leading to their highly disjunctive distribution (e.g. within the genera *Parrya, Campanula, Scutellaria, Dionysia, Silene, Stipa, Viola,* etc.).

### Aridisation of the driest quarter increases the endemism rate

**Figure 7:**
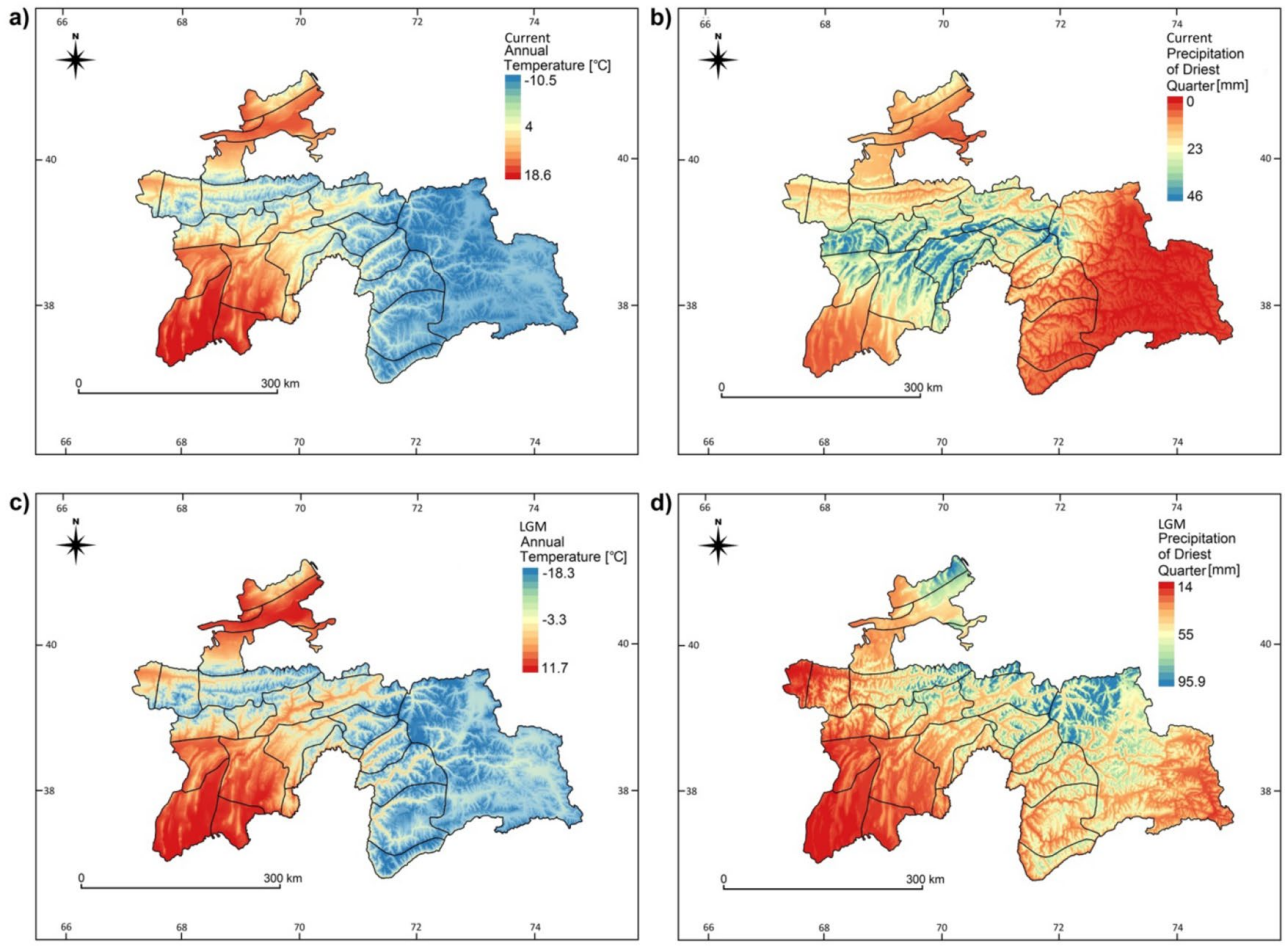
Maps of the most influential climatic variables on the endemism distrution in Tajikistan: current mean annual temperature (**a**), current sum of precipitation of the driest quarter (**b**), mean annual temperature in LGM (**c**), precipitation of the driest quarter in the LGM (**d**). Maps were created using QGIS v. 2.18 (Quantum GIS, https://www.qgis.org).

We found that the precipitation in the driest quarter in the LGM period was an important influential factor for current endemism. The stronger the aridisation of the driest season in the Pleistocene, the higher the endemism proportion today. The pattern is similar to some extent for the current amounts of precipitation in the driest period (summer). The only exceptions were the most arid areas, where the endemic ratio decreases steeply (Figs. 1b, 7b,d). Our findings are similar to those obtained in other regions in the world, particularly in South Africa, California or SW Australia^22^. It has been reported from the regions of Mediterranean type climates, particularly when the summer drought increases and the overall rainfall drops, that aridisation can enhance the ratio of endemic plants in a particular flora^22^. In these arid or semi-arid conditions, several species rich families have been very successful. In the Cape flora, the Asteraceae underwent adaptive radiation during the arid periods of the Miocene and have evolved to include more than a thousand taxa. The second largest family in this extremely species rich region is Fabaceae^23^. A very similar pattern is observed in Tajikistan, where the richest families are exactly the same (Asteraceae—660 species, Fabaceae—520 species). Moreover, these two prominent families encompass the highest number of endemic species, 250 and 297 respectively. This is many more than the next families, the Lamiaceae (98), Apiaceae (77) and Poaceae (over 68)^3^. Within the richest family, the most meaningful genus in terms of endemics is *Astragalus* with 173 endemic taxa. This is the most species rich genus in the world, having its center of diversity in Central and Southwest Asia (Irano-Turanian province). The *Astragalus* spp. is an important element of mountainous and steppe habitats, and its exceptional richness is probably related to niche diversification in the middle to late Pleistocene when environmental conditions in the mountain regions of Southwest and Central Asia cycled repeatedly between dry and moderately humid conditions^5^. The high rate of speciation is probably due to repeated cycles of aridisation of habitats and related drought stress to plants. Although drought stress during part of the vegetation season favors endemic richness, it is clear that the rest of the year must ensure water availability in ecosystems. Extremely dry phytogeographical subregions, such as the eastern part of Prisyrdarian valley and south-west Tajikistan in the county of Shartuz, are the poorest regions both in terms of endemic species and overall diversity of plants.

Our results show that the peak of the endemism rate was found in areas where the difference between the amount of precipitation in the driest quarter of the year in the LGM and today is approx. 30 mm (lower currently). This difference can be assessed as moderate and despite its significance, it is overridden by the energy supply and distribution throughout the year as in other mountainous countries like e.g. the Himalayas^24^.

### The LGM and today’s rain seasonality explain the increase of endemism at extreme values

The results of our study show that low precipitation seasonality in the LGM enhanced contemporary endemism (lower than 40 mm difference). A climate with seasonally evenly distributed rainfall, characteristic for the foothill and colline zones in the LGM, seems likely to favour woodlands that are relatively stable habitats and can harbour support speciation that resulted in a higher rate of current endemism. Probably genera which are rich in endemic species, like *Asyneuma*, *Cousinia*, *Corydalis* and *Ferula,* benefit from this ecosystem. These low stress habitats were particularly suitable for relict Tertiary species like *Ostrowskia magnifica*. On the other hand, the highest amplitudes of precipitation seasonality in the current climate (above 80 mm) prompt the endemism rate in the Tajik flora. Despite the fact that the relation between rain seasonality and endemism can have a highly variable pattern, it is commonly accepted that regions with Mediterranean-like climates with a predictable seasonal cycle, particularly when considering the speciation processes in ancient times, can manifest a high endemism rate^12^. In these endemic-rich areas, the season with rain or mist is highly predictable and totally dry periods of several years do not occur^6^. The uplift of the mountain ranges has considerably differed the precipitation cycles in Central Asia^7^ and created a number of chasmophytic habitats. They were previously empty, but the strong competition in valley bottoms in densely vegetated areas has fostered the “exodus” of plants to rock faces. This was a long-lasting process of adaptation to a harsh environment and nutrient poor habitats. However, probably during the orogenesis and repeated interglacial warming in the Pleistocene, a number of genera revealed their high potential for adaptation to rupiculous habitats. In many taxa, we can find a higher number of species occurring mainly on chasmophytic habitats with only a few congeners in grasslands or shrubby vegetation. The most prominent examples are *Acantholimon* spp., *Asperula* spp., *Draba* ssp., *Kudrjaschevia* ssp., *Parrya* spp., *Pentanema* spp., *Scutellaria* spp., *Silene* spp., and *Viola* spp.^25,26^. All these taxa are well adapted to a highly varied water supply and highly stressful conditions and represent neoendemics in the Tajik flora. It is well documented that stress level can foster specialisation and trait selection^15^. For example, nutrient scarcity poses selective pressure and fosters the selection of new nutritional strategies and evolutionary innovations. Ecologically-stressed environments are associated with the evolution of complex biotic interactions, and are stages for manifold, intriguing co-evolutionary phenomena^27,28^. Examples of this are symbiotic parasitic and mutualistic associations^29^. The beneficial role of arbuscular mycorrhizal symbiosis against abiotic stress factors was ascertained in *Thymus* on calcium and magnesium rich rocks^30^. Other examples from Pamir-Alai include *Asperula pamirica*, *Mathiola integrifolia*, *Spiraea baldshuanica*, *Scutellaria iskanderi*, and other Tajik endemics occurring on the rocks, screes or steppes of the Zeravshan Mts.^31^. Several special nutritional strategies^32–34^ have evolved on nutrient poor bedrocks. These include specialized P-mining roots, some forms of mycorrhiza, insectivory by carnivorous plants, ancient types of plant parasitism, and special forms of N_2_-fixing, e.g.^33–36^. Additionally, adaptive divergent responses to stress explain the recent speciation via the mutation-order mechanism and rapid expansion in gingers^37^.

### Relatively low influence of the yearly precipitation, particularly in current times

Despite the fact that the average yearly precipitation was not the best explanatory factor in relation to the endemism rate, it has a fairly clear peak in areas with ca. 1000 mm in the LGM and ca. 800 mm currently (Figs. 3c, 4c). Combining this result with the data for the driest quarter precipitation in the Pleistocene, we can assume that the rate of endemism is higher when a seasonal drought extreme occurs. However, at the same time the yearly precipitation must be moderate. This typically Mediterranean climate (a kind of palaeo-Mediterranean one) promotes species that are adapted to a seasonally extreme climate with a wet winter/spring and dry summer, and promotes non-woody species, particularly geophytes and annuals^12^. Indeed, the majority of Tajik flora falls into these life-form categories with perennials counting for ca. 58% and annuals ca. 23%^4^. Moreover, within the group of endemics the striking number of Liliaceae (74; 62.7%), Amarylidaceae (43; 51.7%) and Iridaceae (17; 82.1%) is apparent. Additionally, Apiaceae includes a significant number of endemic taxa (77; 43.8%). One of the most valuable and iconic plant groups of Tajikistan are bulbiferous geophytes. Almost thirty species of tulips originated from this country, with 90% of them endemic. Even more diverse is the genus of *Gagea*. Of its 33 species, 13 have the status of national endemics, with *Gagea exilis*, *G. gymnopoda*, *G. holochiton*, *G. incrustata* and *G*. *pseudoerubescens* as the most narrowly distributed. Another ornamental group of geophyte species are foxtail lilies (*Eremurus* sp.). Tajikistan is a core distributional area for 29 *Eremurus* taxa, half of them endemic to Tajikistan. There are several other decorative bulbs with a blooming period in early spring, among them *Juno* (13 species), *Korolkowia* (1 taxon), *Ungernia* (3 species), *Fritillaria* (3 species), *Rhinopetalum* (4 species) being the most prominent. With more than 130 species, the genus *Allium* is one of the most renowned group of taxa that has the centre of its geographical distribution in Central Asia. Many of them are endemic to the country, such as *Allium brevidens*, *A. darvasicum*, *A. komarovii* and many others.

### Strong impact of glacial and current average yearly temperature

A profound effect of energy supply on endemism in the Pamir-Alai Mts. is expressed by the average yearly temperature (Figs. 3a, 4a). Many ecologists agree that energy poses a prominent control in cold climates^17,24^. Our findings are congruent with the results of species richness predictions in neighbouring mountain ranges like the Himalayas^24^. However, we demonstrated the importance of the change of energy supply between the LGM period and current times. The highest endemism rate was found in areas with an increase of the annual average temperature of ca. 10 °C (from − 10 °C in the LGM to the current 0 °C) (Figs. 1b, 7a,c). This considerable change surely influenced productivity and the competition between species. Along with the energy hypothesis, this explains energy partitioning among species. These circumstances are inevitably responsible for increased species richness^38^.

The sensitivity of the endemism rate to energy supply with regard to the LGM period is revealed not only by the significance of the average temperature, but also by its seasonal variation. The greater the variations of monthly means, the lower the endemism rate. Again this is congruent with the results of the current interactions between plant richness and climatic variables in the Western Himalayas. Temperature seasonality was the most powerful predictor of species richness and exhibited a negative correlation^24^. This apparently shows the dependence of the number of endemic species in relation to the whole flora on the stabilisation of the energy supply. This is proved by the proportion of endemic species in the tropics with the highest peaks of endemism in the most stabilised temperature conditions^12^. The phenomenon of increased endemism in more isothermal areas has been reported from mountainous areas in the context of elevation gradient. In relation to temperature, the mountain tops, thus the alpine, subnival and nival belts, are more “ecologically stable” than the mid-altitude or foothill areas. In the Alps, the highest elevations represent annual temperature amplitudes of less than 15ºC whereas colline and montane belts have higher amplitudes. In this sense, the climates of high mountain areas tend to be more stable than those of lower regions, and this favours increased relative endemism^39^. This pattern repeats itself in other mountainous regions with alpine flora rich in local and regional endemics covering overall 3% of the vegetated land area, but including some 4% of all known plant species^39^. This pattern may have a significant influence on endemism patterns in evolutionary time in high-mountain areas, particularly those not fully glaciated.

Considering climatic and energy supply stability, it should also be stressed that the diversified landscape in mountainous areas can also have a facilitating and buffering effect on local flora. The Cenozoic orogenesis introduced to the landscape of Central Asia various microhabitats providing relative environmental stability for climate refugee species, similarly to many other mountainous regions worldwide. For example the high endemic diversity of the Mediterranean Basin flora, including several palaeoendemic plant species, is largely explained by the persistence of the refugial areas in the mountains during glacial periods. Favourable geographical position and high topographical (habitat) variability of the Mediterranean region provided climatically stable areas, where Tertiary (relict) species have evolved and persisted^16^.

## Conclusions

In the era of dramatic climate changes, accurate estimation and understanding of climate-biodiversity relationships will allow us to accurately predict future species richness shifts. Therefore, developing approaches facilitating this task is one of the most important issues which science and conservation planning is facing today.

In our first national-scale model of relationships between climate and plant-species richness patterns in Tajikistan, we evaluated the importance of the Pleistocene, the current climate, and climatic stability in explaining patterns of endemism. According to our results, climatic conditions during the LGM have the greatest influence on contemporary patterns of endemism in Tajikistan. Taking into account individual factors, annual temperature and precipitation fluctuations have been the most crucial drivers of PE. The study revealed the dependence between high PE and a stabilized water-energy supply, something which is undergoing dramatic changes nowadays. On the other hand, we have evidenced that the areas with the highest temperature increase since LGM (above 10 °C) and high average annual contemporary temperatures (above 18 °C) have a low endemism rate.

Due to the low adaptive capacity, the ecosystems of the Pamir-Alai Mts. are particularly sensitive to climate change. They have been already affected by increasing temperatures followed by glacier melting^40^. At the present day, the temperature is the major factor favouring endemism in high, isolated mountain areas ^19^. Therefore, ongoing climate warming in Tajikistan threatens the persistence of isolated populations of unique cryophilous plant species. The process of extinction will touch particularly areas with isolated ecosystems, where the potential escape of plant species is highly limited.

## Material and methods

### Study area

Tajikistan (36°40′–41°05′ E and 67°31′–75°14′ N), with an area of ca. 143 500 km^2^, is a mountainous region with altitudes ranging from 300 to 7500 m a. s. l. Nearly 50% of its territory is above 3000 m a. s. l. and around 93% is over 1000 m a. s. l. The geological structure of the study area is very complex, with outcrops of rocks formed from the Precambrian to the present age. The rocks are generally limestone (micritic, bitumic, marly and dolomitic coral limestone), marble, dolomite, dolomitic shale, clay shale, phyllitic schist, and argillaceous slate. Several kinds of metamorphic rocks are also present within the study area (cf. Nedzvedskiy^41^).

Tajikistan has a generally high level of solar insolation (2,090–3,160 sunshine hours), a low percentage of cloud cover, high-amplitude annual temperatures, and moderate humidity and precipitation (with the exception of the spring period). The large-scale precipitation dynamic is shaped by the influence of west and south-west cyclones during spring and Siberian Anticyclone during winter^7^. Annual precipitation in western Pamir-Alai ranges from ca. 350 mm (Zeravshan Mts.) to ca. 600 mm in the Hissar Range (in some locations up to 2000 mm). The lower bound of permanent snow is from 3500 to 3600 m a. s. l. in the western part to 5800 m a. s. l. in the eastern part of the country^2,42,43^. Temperature variation is mainly dependent on altitude and radiation balance^7^. In the alpine belt of high mountains, the climate is more harsh, with average temperatures in July between 9.7 °C and 13.5 °C. According to bioclimatic classification which takes into consideration mainly precipitation and temperature values, the study area could be classified as the Mediterranean type of macrobioclimate^44^. Summer droughts last for at least two consecutive months in which P < 2 T (the annual precipitation value less than twice the annual temperature value), the yearly average temperature is below 25 °C (ca. 2 °C), the Compensated Thermicity Index is below 580 (ca. 80), and the Continentality Index is ca. 31. Tajikistan fits, therefore, the continental type (eucontinental subtype), and recent research on the SW and Central Asia bioclimate suggests that the Irano-Turanian bioclimatic zone should be distinguished by higher continentality, lower precipitation (particularly during winter), a longer dry season and lower winter temperature minima when compared to the Mediterranean bioclimate. Tajik climate is also distinct from the Central Asiatic climate because of its lower and unequal precipitation (with an apparent spring peak), drier summer season and lower continentality^45^.

### Tajik flora: data collection

The dataset used in this study contains all known vascular plant species stations in Tajikistan reported in the 10-volume work dedicated to the flora of the former Soviet Socialist Republic of Tajikistan, prepared by a multiauthor team (^46–54^; and supplemented by a few other authors) that lists approx. 4,300 species. These species are assigned to 116 families and 994 genera. For this study, we collected the following information about each species: (1) its presence and absence in a subregion; (2) its presence and absence in an elevation range at every 100 m band, which is the maximum precision reported in the flora of Tajikistan.

To show the chorological pattern of vascular plant endemism, we used the system of operational geographic units (OGUs) defined by phytogeography and elevation. Each unit thus represents a polygon of phytogeographical subregion (Fig. 1a) according to the division proposed by Grubov^55^ and a 100-m elevation belt. In this way, we obtained 808 OGUs with relatively homogeneous environmental parameters (Appendix S1). Using the available literature, on the basis of presence/absence data, we compiled all plant species elevation distributional ranges for each phytogeographical subregion in Tajikistan. Then, we assigned information about potential plant species richness and endemic species richness for each OGU. We obtained 336 749 records’ matrix. The elevations above 5100 in East Tajikistanian A, B and C, Alaian and Zeravshanian C, and above 5700 in West Pamirian A, B and C, East Pamirian were omitted because no vascular plants occur at those altitude ranges.

### Input data

We compiled a map of elevation ranges in phytogeographical subregions based on the digital elevation model (DEM) with ca. 100 m resolution^56^. To each OGU, based on species richness data, we attributed information about plant endemism (proportion of endemic plant species to overall plant species richness—PE; Fig. 1b). Accordingly, we assigned mean values of contemporary and glacial (from the Last Glacial Maximum, LGM) climatic characteristics, which were derived from the CHELSA dataset, with a resolution of ca. 1 km × 1 km^57^. The information about LGM climate was derived from the MICROC_ESM model. The datasets contained 19 bioclimatic variables^57^ each. All maps were handled using QGIS v. 2.18 (Quantum GIS, https://www.qgis.org) and Saga GIS open source software (Appendix S2). Accordingly, to assess climate stability, we calculated the difference between variables representing the current and LGM climate for each OGU.

### Statistical analyses

To explore the potential causal explanations of the current climate, LGM climate and climate stability for the patterns of PEs, we performed a generalized additive model (GAM) that allowed for the fitting of a nonlinear curve without a priori specification of its functional form^58^. Prior to analysis, bioclimatic variables were selected based on a Pearson correlation analysis. Variables indicating strong multicollinearity (r > 0.7 all pairwise comparisons) were removed, and we retained variables that were ecologically more relevant predictors^59^. The variables used for modeling were: bio1—annual mean temperature, bio3—isothermality, bio12—annual precipitation, bio15—precipitation seasonality, and bio17—precipitation of the driest quarter. Next, we built three GAMs including all the retained bioclimatic variables, the first with current climate variables, the second with LGM climate variables, and the third with climate stability variables included. In each model, smoothing was performed automatically with cubic regression splines^60^. Cubic splines were applied to all predictors.

We used a regression tree approach applying the R package *rpart*^61^ to determine the multivariate relationships among PE and selected climatic variables. Classification and regression trees are often used to construct predictive models with biological data, and they can also be used to show a simple descriptive structure for complex data^62^. These trees are flexible non-parametric multivariate analyses that provide dichotomous keys for each bioclimatic variable, which may involve complex interactions. We conducted four classification-tree analyses. The first three analyses were separate classifications calculated for each group of climatic predictors (current climate, LGM climate and climate stability). The last regression tree model included all predictors, to indicate the most important bioclimatic variables for shaping the PEs.

## Supplementary Information

Supplementary Information.

## Data Availability

The raw data that support the findings of this study are available from the corresponding author upon request.
