# Supplementary material for: Palaeoclimate has a major effect on the diversity of endemic species in the hotspot of mountain biodiversity in Tajikistan
Source: Sci Rep. 2021 Sep 21;11:18684. doi: 10.1038/s41598-021-98027-3 (PMC8455614; doi:10.1038/s41598-021-98027-3)
Supplement: Supplementary file 1 — Supplementary Information. [file 41598_2021_98027_MOESM1_ESM.docx]

**Table S1.** Descriptive statistics (mean and standard deviation) concerning species diversity and altitude for 26 subregions of Tajikistan

|  |  | Species richness | |  | Endemic species richness | |  | Plant endemism* | |  | Altitude  [m. a. s. l.] | |
| --- | --- | --- | --- | --- | --- | --- | --- | --- | --- | --- | --- | --- |
|  |  | M | SD |  | M | SD |  | M | SD |  | M | SD |
| 1 | Alaian | 71.1 | 37.4 |  | 5.6 | 3.4 |  | 0.07 | 0.04 |  | 3550 | 823 |
| 2 | East Pamiraian | 308.1 | 239.8 |  | 50.5 | 39.5 |  | 0.13 | 0.07 |  | 4150 | 880 |
| 3 | East Tajikistan A | 405.4 | 231.4 |  | 62.1 | 38.2 |  | 0.14 | 0.03 |  | 3200 | 1082 |
| 4 | East Tajikistan B | 317.4 | 200.2 |  | 53.8 | 36.9 |  | 0.15 | 0.05 |  | 3400 | 967 |
| 5 | East Tajikistan C | 261.3 | 160.9 |  | 37.0 | 26.5 |  | 0.11 | 0.05 |  | 3250 | 1054 |
| 6 | Hissar-Darvasian A | 627.1 | 276.4 |  | 112.8 | 57.1 |  | 0.17 | 0.04 |  | 2550 | 1054 |
| 7 | Hissar-Darvasian B | 402.7 | 206.9 |  | 72.1 | 42.3 |  | 0.16 | 0.05 |  | 2800 | 1025 |
| 8 | Hissar-Darvasian C | 395.8 | 168.7 |  | 67.9 | 33.0 |  | 0.17 | 0.03 |  | 2600 | 1025 |
| 9 | Hissar-Darvasian D | 537.7 | 264.0 |  | 99.0 | 58.7 |  | 0.17 | 0.04 |  | 2650 | 1111 |
| 10 | Hissar-Darvasian E | 274.7 | 179.0 |  | 50.8 | 36.5 |  | 0.16 | 0.06 |  | 2950 | 1227 |
| 11 | Hissar-Darvasian F | 533.3 | 128.8 |  | 98.3 | 33.2 |  | 0.18 | 0.03 |  | 1650 | 707 |
| 12 | Kuraminian | 351.8 | 136.0 |  | 21.3 | 12.2 |  | 0.05 | 0.02 |  | 2000 | 1025 |
| 13 | Mogoltausian | 375.6 | 111.8 |  | 22.4 | 9.2 |  | 0.06 | 0.01 |  | 950 | 418 |
| 14 | Prisyrdarian | 528.7 | 92.9 |  | 21.3 | 4.0 |  | 0.04 | 0.00 |  | 1050 | 476 |
| 15 | South Tajikistan A | 836.8 | 141.9 |  | 115.0 | 33.0 |  | 0.14 | 0.03 |  | 1350 | 534 |
| 16 | South Tajikistan B | 735.9 | 218.5 |  | 123.5 | 49.8 |  | 0.16 | 0.04 |  | 1750 | 823 |
| 17 | South Tajikistan C | 739.3 | 154.2 |  | 97.5 | 29.8 |  | 0.13 | 0.02 |  | 1050 | 476 |
| 18 | South Tajikistan D | 501.5 | 131.4 |  | 66.8 | 24.1 |  | 0.13 | 0.03 |  | 1000 | 447 |
| 19 | Turkestanian A | 412.9 | 214.4 |  | 39.2 | 27.8 |  | 0.08 | 0.04 |  | 2600 | 1256 |
| 20 | Turkestanian B | 335.5 | 42.2 |  | 26.3 | 8.8 |  | 0.08 | 0.02 |  | 1850 | 707 |
| 21 | West Pamirian A | 201.5 | 146.9 |  | 27.0 | 20.9 |  | 0.10 | 0.06 |  | 3555 | 1208 |
| 22 | West Pamirian B | 363.1 | 272.2 |  | 58.7 | 42.8 |  | 0.14 | 0.07 |  | 3817 | 1050 |
| 23 | West Pamirian C | 294.5 | 227.1 |  | 44.1 | 32.7 |  | 0.13 | 0.07 |  | 3900 | 1025 |
| 24 | Zeravshanian A | 410.2 | 211.0 |  | 52.7 | 31.9 |  | 0.13 | 0.04 |  | 2600 | 1082 |
| 25 | Zeravshanian B | 564.6 | 365.1 |  | 85.5 | 64.5 |  | 0.13 | 0.06 |  | 3000 | 1198 |
| 26 | Zeravshanian C | 516.0 | 332.6 |  | 82.6 | 60.2 |  | 0.14 | 0.05 |  | 3200 | 1082 |

* proportion of endemic plant species to overall plant species richness

**Table S2.** Descriptive statistics (mean and standard deviation) concerning current climatic variables used in the analysis for 26 subregions of Tajikistan

|  |  | Mean Annual Temperature [°C] | |  | Isothermality | |  | Sum of Annual Precipitations  [mm] | |  | Precipitation Seasonality | |  | Sum of Precipitation in Driest Quarter [mm] | |
| --- | --- | --- | --- | --- | --- | --- | --- | --- | --- | --- | --- | --- | --- | --- | --- |
|  |  | M | SD |  | M | SD |  | M | SD |  | M | SD |  | M | SD |
| 1 | Alaian | -2.0 | 4.0 |  | 29.5 | 0.0 |  | 542.2 | 105.9 |  | 52.3 | 0.5 |  | 32.4 | 7.7 |
| 2 | East Pamiraian | -5.7 | 3.8 |  | 29.0 | 0.3 |  | 217.0 | 87.8 |  | 42.9 | 4.3 |  | 19.3 | 7.7 |
| 3 | East Tajikistan A | 0.2 | 5.6 |  | 29.0 | 0.2 |  | 609.2 | 112.4 |  | 51.6 | 2.6 |  | 30.4 | 7.0 |
| 4 | East Tajikistan B | -0.8 | 4.8 |  | 28.7 | 0.2 |  | 619.1 | 126.3 |  | 56.0 | 0.8 |  | 22.6 | 6.0 |
| 5 | East Tajikistan C | -0.5 | 5.0 |  | 28.6 | 0.3 |  | 396.8 | 167.6 |  | 58.8 | 2.7 |  | 15.1 | 7.7 |
| 6 | Hissar-Darvasian A | 4.8 | 5.9 |  | 29.0 | 0.1 |  | 819.4 | 39.9 |  | 68.9 | 3.7 |  | 24.5 | 6.2 |
| 7 | Hissar-Darvasian B | 3.3 | 5.5 |  | 29.3 | 0.1 |  | 705.3 | 85.9 |  | 69.6 | 2.9 |  | 22.4 | 7.5 |
| 8 | Hissar-Darvasian C | 4.1 | 5.5 |  | 29.4 | 0.1 |  | 632.7 | 60.0 |  | 63.5 | 3.3 |  | 23.9 | 7.6 |
| 9 | Hissar-Darvasian D | 4.1 | 6.0 |  | 30.0 | 0.6 |  | 827.7 | 140.2 |  | 70.2 | 7.2 |  | 16.3 | 6.3 |
| 10 | Hissar-Darvasian E | 2.2 | 6.2 |  | 29.8 | 0.5 |  | 617.8 | 136.6 |  | 67.7 | 4.0 |  | 11.9 | 4.5 |
| 11 | Hissar-Darvasian F | 9.9 | 3.7 |  | 31.3 | 0.0 |  | 660.8 | 179.3 |  | 85.0 | 0.6 |  | 4.8 | 1.5 |
| 12 | Kuraminian | 5.9 | 6.0 |  | 28.7 | 0.2 |  | 478.4 | 107.6 |  | 62.0 | 2.9 |  | 16.0 | 7.4 |
| 13 | Mogoltausian | 12.6 | 2.2 |  | 29.5 | 0.0 |  | 375.6 | 33.1 |  | 66.9 | 0.6 |  | 7.8 | 0.6 |
| 14 | Prisyrdarian | 11.9 | 2.6 |  | 29.0 | 0.2 |  | 268.3 | 17.9 |  | 55.9 | 1.9 |  | 13.3 | 2.3 |
| 15 | South Tajikistan A | 12.2 | 2.9 |  | 29.2 | 0.2 |  | 633.8 | 90.7 |  | 79.2 | 2.3 |  | 9.8 | 2.5 |
| 16 | South Tajikistan B | 9.8 | 4.7 |  | 30.2 | 0.1 |  | 816.3 | 202.8 |  | 80.5 | 3.8 |  | 10.7 | 5.7 |
| 17 | South Tajikistan C | 14.3 | 3.0 |  | 29.0 | 0.2 |  | 322.2 | 67.1 |  | 89.6 | 1.3 |  | 1.6 | 0.8 |
| 18 | South Tajikistan D | 14.5 | 2.7 |  | 30.2 | 0.2 |  | 400.6 | 67.8 |  | 89.3 | 0.7 |  | 1.0 | 0.4 |
| 19 | Turkestanian A | 3.4 | 6.6 |  | 30.2 | 0.1 |  | 406.9 | 63.7 |  | 59.0 | 2.9 |  | 28.3 | 7.3 |
| 20 | Turkestanian B | 7.6 | 3.6 |  | 29.6 | 0.2 |  | 270.2 | 63.5 |  | 50.1 | 0.4 |  | 25.9 | 8.1 |
| 21 | West Pamirian A | -2.2 | 5.6 |  | 28.7 | 0.5 |  | 250.8 | 66.9 |  | 62.3 | 5.0 |  | 8.9 | 5.1 |
| 22 | West Pamirian B | -3.5 | 4.8 |  | 29.1 | 0.5 |  | 182.4 | 24.8 |  | 68.2 | 4.1 |  | 6.0 | 3.0 |
| 23 | West Pamirian C | -3.7 | 4.6 |  | 29.1 | 0.4 |  | 162.7 | 35.2 |  | 76.2 | 2.9 |  | 5.5 | 2.3 |
| 24 | Zeravshanian A | 3.7 | 5.7 |  | 30.9 | 0.2 |  | 531.7 | 90.3 |  | 63.7 | 2.7 |  | 23.9 | 6.8 |
| 25 | Zeravshanian B | 1.6 | 6.4 |  | 29.8 | 0.3 |  | 483.5 | 88.9 |  | 60.5 | 0.9 |  | 24.4 | 5.0 |
| 26 | Zeravshanian C | 0.4 | 5.7 |  | 29.5 | 0.1 |  | 433.9 | 73.5 |  | 57.4 | 1.8 |  | 30.7 | 7.1 |

**Table S3.** Descriptive statistics (mean and standard deviation) concerning glacial climatic variables used in the analysis for 26 subregions of Tajikistan

|  |  | Mean Annual Temperature  [°C] | |  | Isothermality | |  | Sum of Annual Precipitations [mm] | |  | Precipitation Seasonality | |  | Sum of Precipitation in Driest Quarter [mm] | |
| --- | --- | --- | --- | --- | --- | --- | --- | --- | --- | --- | --- | --- | --- | --- | --- |
|  |  | M | SD |  | M | SD |  | M | SD |  | M | SD |  | M | SD |
| 1 | Alaian | -8.3 | 5.1 |  | 29.1 | 0.0 |  | 801.6 | 198.5 |  | 52.6 | 1.5 |  | 79.1 | 23.8 |
| 2 | East Pamiraian | -10.9 | 5.1 |  | 29.2 | 0.1 |  | 397.3 | 104.6 |  | 41.6 | 1.5 |  | 55.9 | 18.8 |
| 3 | East Tajikistan A | -5.7 | 7.0 |  | 29.2 | 0.0 |  | 645.8 | 107.0 |  | 54.1 | 2.4 |  | 62.0 | 15.4 |
| 4 | East Tajikistan B | -6.8 | 6.1 |  | 29.2 | 0.0 |  | 589.8 | 108.3 |  | 55.6 | 1.7 |  | 52.9 | 14.0 |
| 5 | East Tajikistan C | -5.0 | 6.7 |  | 29.3 | 0.0 |  | 467.2 | 101.3 |  | 54.0 | 2.5 |  | 44.0 | 13.3 |
| 6 | Hissar-Darvasian A | -3.8 | 7.0 |  | 30.0 | 0.0 |  | 822.1 | 185.9 |  | 67.9 | 3.0 |  | 34.9 | 9.9 |
| 7 | Hissar-Darvasian B | -5.0 | 6.6 |  | 29.6 | 0.0 |  | 895.3 | 239.7 |  | 67.1 | 2.1 |  | 51.2 | 17.3 |
| 8 | Hissar-Darvasian C | -3.0 | 6.8 |  | 29.4 | 0.0 |  | 806.9 | 236.1 |  | 63.8 | 1.7 |  | 55.2 | 17.9 |
| 9 | Hissar-Darvasian D | -2.7 | 6.9 |  | 29.4 | 0.1 |  | 685.0 | 160.8 |  | 64.4 | 2.0 |  | 47.7 | 17.0 |
| 10 | Hissar-Darvasian E | -4.0 | 7.6 |  | 29.4 | 0.0 |  | 643.7 | 161.1 |  | 62.2 | 2.3 |  | 48.0 | 16.4 |
| 11 | Hissar-Darvasian F | 3.8 | 4.5 |  | 29.6 | 0.0 |  | 549.0 | 132.5 |  | 70.5 | 0.7 |  | 29.7 | 6.8 |
| 12 | Kuraminian | -0.5 | 6.6 |  | 29.2 | 0.1 |  | 900.1 | 213.2 |  | 52.2 | 1.6 |  | 76.0 | 25.4 |
| 13 | Mogoltausian | 6.9 | 2.4 |  | 29.4 | 0.0 |  | 769.6 | 105.7 |  | 58.7 | 0.8 |  | 52.8 | 7.5 |
| 14 | Prisyrdarian | 6.6 | 2.9 |  | 29.2 | 0.0 |  | 742.7 | 102.9 |  | 56.9 | 1.6 |  | 63.7 | 10.0 |
| 15 | South Tajikistan A | 4.8 | 3.3 |  | 30.1 | 0.0 |  | 649.7 | 119.9 |  | 72.7 | 1.6 |  | 25.3 | 4.5 |
| 16 | South Tajikistan B | 2.7 | 5.3 |  | 29.6 | 0.1 |  | 699.3 | 179.8 |  | 68.5 | 1.4 |  | 36.9 | 12.1 |
| 17 | South Tajikistan C | 7.0 | 3.3 |  | 30.2 | 0.0 |  | 585.6 | 107.8 |  | 75.6 | 0.5 |  | 20.6 | 4.0 |
| 18 | South Tajikistan D | 7.6 | 3.0 |  | 29.9 | 0.0 |  | 583.9 | 110.2 |  | 76.1 | 1.0 |  | 25.9 | 5.2 |
| 19 | Turkestanian A | -4.3 | 8.0 |  | 29.7 | 0.1 |  | 793.8 | 228.5 |  | 66.7 | 1.9 |  | 46.0 | 13.4 |
| 20 | Turkestanian B | 2.4 | 4.2 |  | 29.2 | 0.0 |  | 620.4 | 61.3 |  | 56.2 | 1.7 |  | 58.1 | 4.7 |
| 21 | West Pamirian A | -7.1 | 7.5 |  | 29.4 | 0.0 |  | 429.4 | 84.3 |  | 52.8 | 4.7 |  | 44.6 | 16.3 |
| 22 | West Pamirian B | -9.3 | 6.6 |  | 29.5 | 0.1 |  | 389.3 | 70.8 |  | 53.3 | 5.3 |  | 40.9 | 14.5 |
| 23 | West Pamirian C | -10.7 | 6.6 |  | 29.5 | 0.1 |  | 395.2 | 78.0 |  | 53.1 | 5.2 |  | 42.9 | 14.7 |
| 24 | Zeravshanian A | -4.6 | 6.9 |  | 30.4 | 0.0 |  | 630.2 | 173.4 |  | 70.0 | 2.1 |  | 22.6 | 6.7 |
| 25 | Zeravshanian B | -7.3 | 7.8 |  | 30.1 | 0.0 |  | 763.2 | 232.1 |  | 70.5 | 3.6 |  | 33.3 | 12.3 |
| 26 | Zeravshanian C | -8.0 | 6.5 |  | 29.6 | 0.2 |  | 865.1 | 244.2 |  | 64.8 | 1.1 |  | 56.5 | 26.5 |

**Table S4.** Descriptive statistics (mean and standard deviation) concerning climatic stability variables (climatic changes since the Last Glacial Maximum) used in the analysis for 26 subregions of Tajikistan

|  |  | Mean Annual Temperature  [°C] | |  | Isothermality | |  | Sum of Annual Precipitations  [mm] | |  | Precipitation Seasonality | |  | Sum of Precipitation in the Driest Quarter [mm] | |
| --- | --- | --- | --- | --- | --- | --- | --- | --- | --- | --- | --- | --- | --- | --- | --- |
|  |  | M | SD |  | M | SD |  | M | SD |  | M | SD |  | M | SD |
| 1 | Alaian | 6.3 | 1.1 |  | 0.4 | 0.0 |  | -259.4 | 95.6 |  | -0.3 | 1.3 |  | -46.7 | 16.1 |
| 2 | East Pamiraian | 5.2 | 1.5 |  | -0.3 | 0.3 |  | -180.3 | 44.9 |  | 1.3 | 5.5 |  | -36.5 | 11.7 |
| 3 | East Tajikistan A | 5.8 | 1.3 |  | -0.1 | 0.2 |  | -36.6 | 36.3 |  | -2.4 | 0.7 |  | -31.6 | 8.5 |
| 4 | East Tajikistan B | 6.0 | 1.3 |  | -0.5 | 0.2 |  | 29.2 | 20.2 |  | 0.4 | 1.0 |  | -30.2 | 7.9 |
| 5 | East Tajikistan C | 4.6 | 1.8 |  | -0.7 | 0.3 |  | -70.4 | 66.9 |  | 4.8 | 0.8 |  | -29.0 | 5.6 |
| 6 | Hissar-Darvasian A | 8.6 | 1.1 |  | -1.0 | 0.1 |  | -2.7 | 158.5 |  | 1.0 | 6.4 |  | -10.4 | 3.8 |
| 7 | Hissar-Darvasian B | 8.3 | 1.1 |  | -0.3 | 0.1 |  | -190.0 | 175.8 |  | 2.5 | 4.7 |  | -28.8 | 9.9 |
| 8 | Hissar-Darvasian C | 7.1 | 1.3 |  | 0.0 | 0.1 |  | -174.1 | 200.9 |  | -0.3 | 2.8 |  | -31.3 | 10.4 |
| 9 | Hissar-Darvasian D | 6.7 | 0.9 |  | 0.6 | 0.5 |  | 142.7 | 86.5 |  | 5.8 | 5.3 |  | -31.3 | 11.1 |
| 10 | Hissar-Darvasian E | 6.2 | 1.5 |  | 0.5 | 0.5 |  | -25.9 | 121.3 |  | 5.4 | 1.9 |  | -36.1 | 12.4 |
| 11 | Hissar-Darvasian F | 6.1 | 0.8 |  | 1.7 | 0.0 |  | 111.8 | 50.2 |  | 14.5 | 0.9 |  | -24.9 | 5.3 |
| 12 | Kuraminian | 6.4 | 0.6 |  | -0.5 | 0.1 |  | -421.7 | 114.1 |  | 9.8 | 1.5 |  | -60.0 | 18.2 |
| 13 | Mogoltausian | 5.7 | 0.2 |  | 0.1 | 0.0 |  | -393.9 | 73.0 |  | 8.3 | 0.4 |  | -44.9 | 7.0 |
| 14 | Prisyrdarian | 5.3 | 0.3 |  | -0.2 | 0.2 |  | -474.4 | 86.5 |  | -1.0 | 2.7 |  | -50.4 | 8.5 |
| 15 | South Tajikistan A | 7.4 | 0.4 |  | -0.9 | 0.2 |  | -16.0 | 109.2 |  | 6.6 | 1.0 |  | -15.5 | 5.3 |
| 16 | South Tajikistan B | 7.0 | 0.6 |  | 0.6 | 0.1 |  | 116.9 | 43.7 |  | 12.0 | 3.1 |  | -26.1 | 6.5 |
| 17 | South Tajikistan C | 7.3 | 0.3 |  | -1.2 | 0.3 |  | -263.4 | 44.1 |  | 14.0 | 1.1 |  | -19.0 | 3.3 |
| 18 | South Tajikistan D | 6.9 | 0.3 |  | 0.3 | 0.2 |  | -183.4 | 46.4 |  | 13.2 | 1.0 |  | -24.8 | 4.9 |
| 19 | Turkestanian A | 7.7 | 1.4 |  | 0.5 | 0.2 |  | -386.9 | 172.5 |  | -7.6 | 4.1 |  | -17.7 | 8.5 |
| 20 | Turkestanian B | 5.2 | 0.6 |  | 0.5 | 0.2 |  | -350.2 | 44.9 |  | -6.1 | 1.7 |  | -32.2 | 6.1 |
| 21 | West Pamirian A | 4.9 | 1.9 |  | -0.7 | 0.5 |  | -178.5 | 21.5 |  | 9.5 | 0.6 |  | -35.7 | 11.2 |
| 22 | West Pamirian B | 5.8 | 1.9 |  | -0.4 | 0.4 |  | -206.9 | 46.7 |  | 14.9 | 1.9 |  | -34.9 | 11.5 |
| 23 | West Pamirian C | 7.0 | 2.0 |  | -0.4 | 0.3 |  | -232.5 | 44.9 |  | 23.0 | 2.6 |  | -37.4 | 12.3 |
| 24 | Zeravshanian A | 8.3 | 1.2 |  | 0.4 | 0.2 |  | -98.5 | 92.7 |  | -6.3 | 4.5 |  | 1.3 | 2.9 |
| 25 | Zeravshanian B | 8.9 | 1.4 |  | -0.4 | 0.3 |  | -279.8 | 155.6 |  | -10.0 | 3.5 |  | -8.9 | 8.2 |
| 26 | Zeravshanian C | 8.4 | 0.8 |  | 0.0 | 0.1 |  | -431.2 | 185.1 |  | -7.4 | 2.7 |  | -25.8 | 19.8 |

| 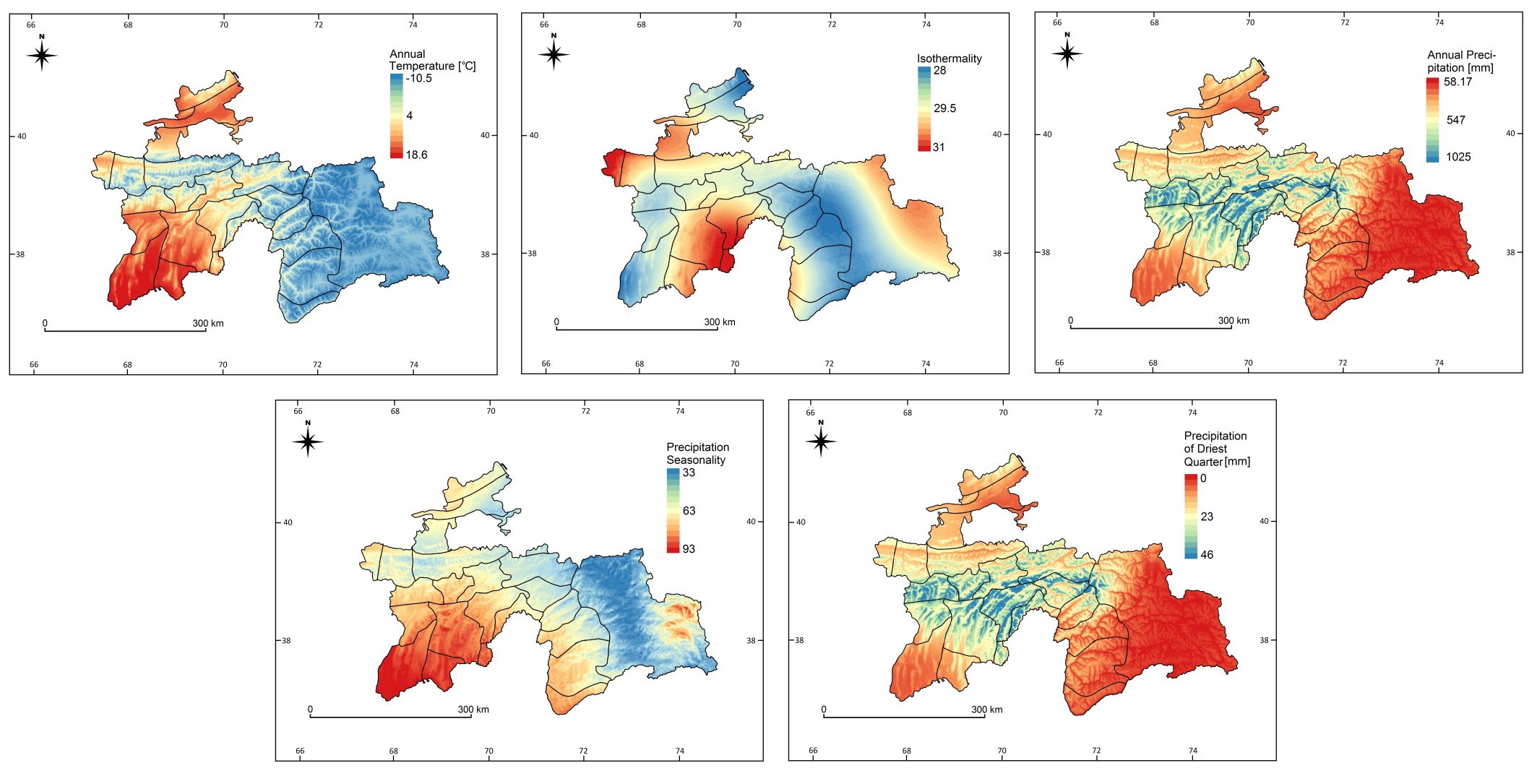 |
| --- |
| **Figure S1.** Maps of current climatic variables used in the analysis. Maps were created using QGIS v. 2.18 (Quantum GIS, https://www.qgis.org). |

| 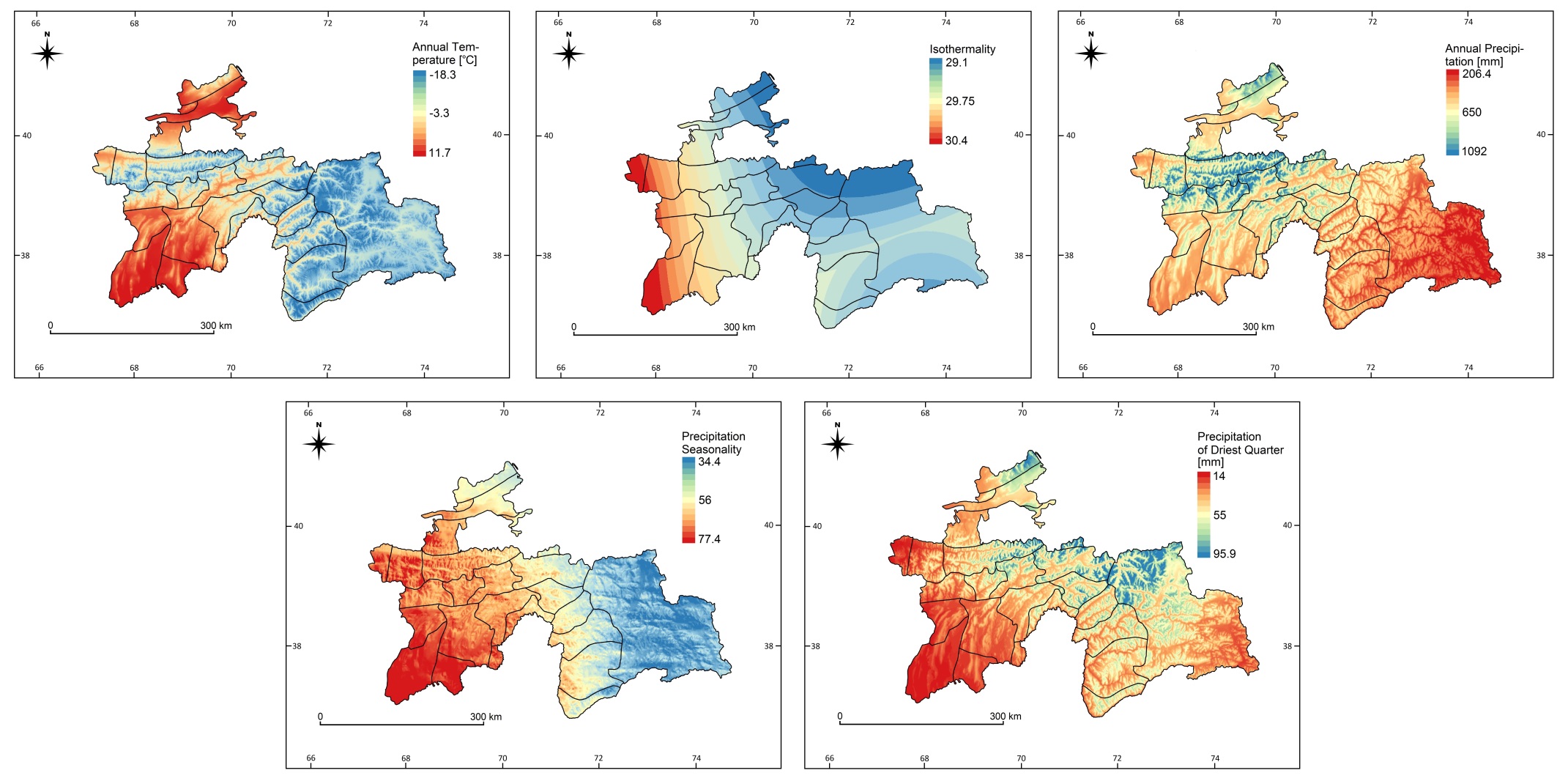 |
| --- |
| **Figure S2.** Maps of LGM climatic variables used in the analysis. Maps were created using QGIS v. 2.18 (Quantum GIS, https://www.qgis.org). |

| 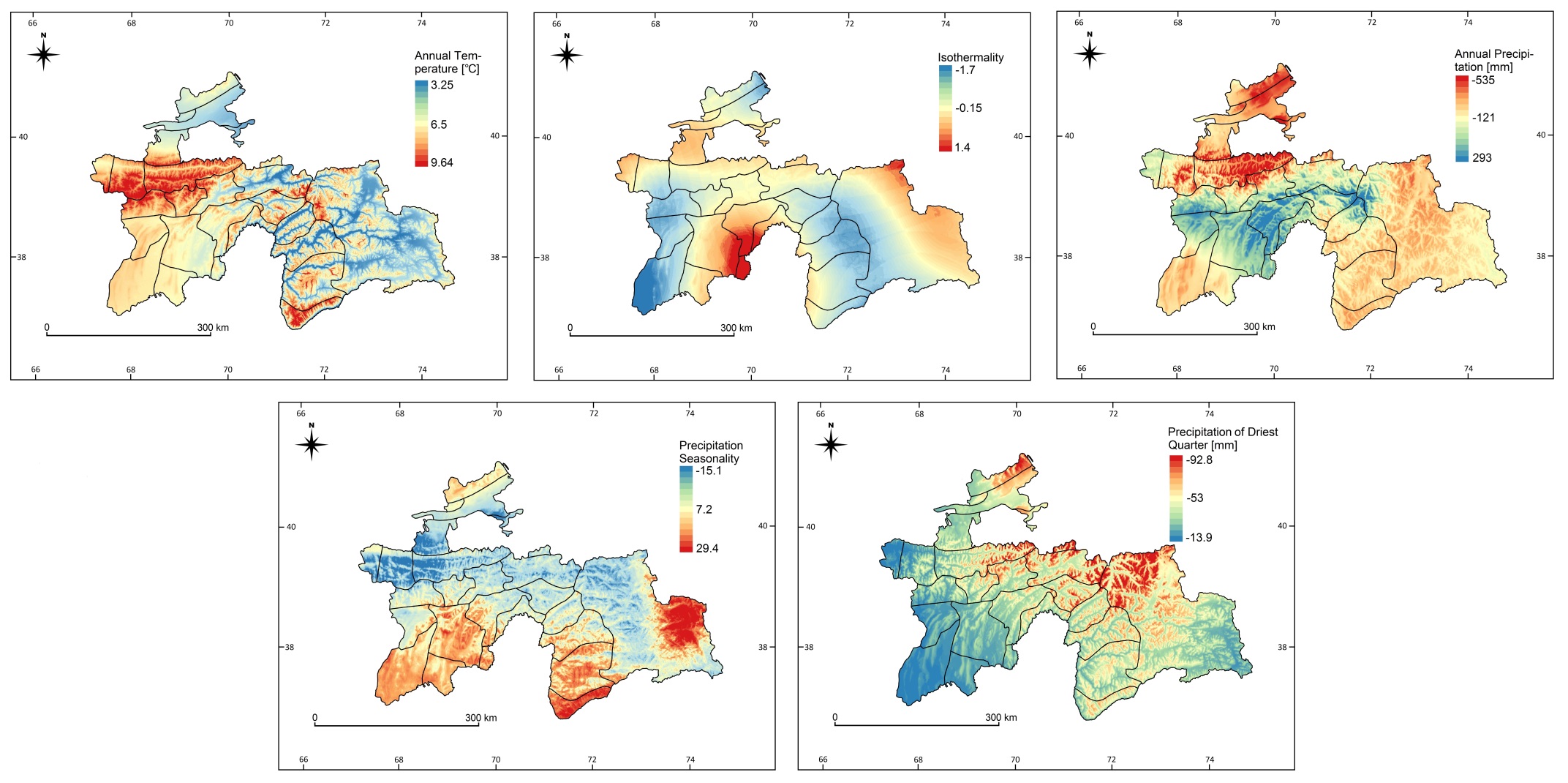 |
| --- |
| **Figure S3.** Maps of variables representing climate stability used in the analysis. Maps were created using QGIS v. 2.18 (Quantum GIS, https://www.qgis.org). |
